# Switching from monoculture to polyculture farming benefits birds in oil palm production landscapes: Evidence from mist netting data

**DOI:** 10.1002/ece3.3205

**Published:** 2017-07-05

**Authors:** Muhammad S. Yahya, Muhamad Syafiq, Adham Ashton‐Butt, Amal Ghazali, Siti Asmah, Badrul Azhar

**Affiliations:** ^1^ Department of Forest Management Faculty of Forestry Universiti Putra Malaysia Selangor Malaysia; ^2^ Institute for Life Sciences University of Southampton Southampton UK; ^3^ Biodiversity Unit Institute of Bioscience Universiti Putra Malaysia Selangor Malaysia

**Keywords:** agriculture, biodiversity, bird, conservation, oil palm, smallholdings

## Abstract

Monoculture farming is pervasive in industrial oil palm agriculture, including those RSPO plantations certified as sustainably managed. This farming practice does not promote the maintenance of farmland biodiversity. However, little scientific attention has been given to polyculture farming in oil palm production landscapes. Polyculture farming is likely to increase the floristic diversity and stand structural complexity that underpins biodiversity. Mist nets were used to sample birds at 120 smallholdings in Peninsular Malaysia. At each site, 12 vegetation structure characteristics were measured. We compared bird species richness, abundance, and composition between monoculture and polyculture smallholdings and used predictive models to examine the effects of habitat quality on avian biodiversity. Bird species richness was significantly greater in polyculture than that of monoculture smallholdings. The number of fallen and standing, dead oil palms were also important positive predictors of species richness. Bird abundance was also strongly increased by standing and dead oil palms and decreased with oil palm stand height. Our results indicate that polyculture farming can improve bird species richness in oil palm production landscapes. In addition, key habitat variables that are closely associated with farming practices, such as the removal of dead trees, should and can be managed by oil palm growers in order to promote biodiversity. To increase the sustainability of oil palm agriculture, it is imperative that stakeholders modify the way oil palms are currently planted and managed. Our findings can guide policy makers and certification bodies to promote oil palm production landscapes that will function more sustainably and increase existing biodiversity of oil palm landscapes.

## 1 INTRODUCTION

Oil palm agriculture is rapidly expanding worldwide at the expense of tropical rainforests (Härdter, Chow, & Hock, 1997; Fitzherbert et al., 2008; Wilcove & Koh, 2010). Strong global demands for inexpensive vegetable oil from emerging economic powers such as China and India have contributed to the expansion of commercial oil palm cultivation (Koh & Wilcove, 2007). The land area planted with oil palm is increasing in South America, western Africa, and Southeast Asia (FAOSTAT 2016). Forest conversion to oil palm is widely documented and has been estimated at 270,000 ha per year in major oil palm producing countries (Henders, Persson, & Kastner, 2015). However, forest conversion to monoculture plantations is not the only way which oil palm agriculture has expanded. Conversion from other commodity, perennial crops also has taken place because of government incentives and market demand (Basiron, 2007). In Southeast Asia, oil palm is replacing rubber, coconut, and cacao (Feintrenie, Chong, & Levang, 2010; Wicke, Sikkema, Dornburg, & Faaij, 2011). This crop‐based conversion is poorly understood compared to the much publicized deforestation‐based conversion. Because of this, the palm oil industry is always linked to tropical deforestation and biodiversity loss in producing countries.

In the wake of intense, antipalm oil campaigns organized by environmental NGOs, palm oil stakeholders are under pressure to improve their environmental performance, for example by establishing a zero‐deforestation policy in the establishment of new plantations (Corley, 2009; Khor, 2011). Thus, improving biodiversity in oil palm landscapes has become a key management policy to palm oil stakeholders to mitigate the negative effects of palm oil production on biodiversity. Besides maintaining critical habitats such as forest patches and riparian habitats (Gray, Slade, Mann, & Lewis, 2014; Lucey et al., 2014), other management options such as controlling illegal hunting, road accidents, increasing noncrop floral diversity, and tree‐based enrichment have been suggested to reconcile biodiversity conservation and palm oil production (Azhar et al., 2013; Teuscher et al., 2016). To achieve this, evidence‐based management options are being investigated by conservation scientists (Foster et al., 2011). However, the options are limited and not directly related to oil palm agronomy. To date, commercial oil palm agriculture is dominated by monoculture systems (Azhar, Puan, et al., 2015; Azhar, Saadun, et al., 2015). Globally, this oil palm production system characterizes large‐scale plantations and some smallholdings (Azhar, Puan, Zakaria, Hassan, & Arif, 2014). The impacts of oil palm monoculture systems on biodiversity have been studied extensively, particularly for some animal taxa such as birds and amphibians (Aratrakorn, Thunhikorn, & Donald, 2006; Edwards et al., 2010; Gallmetzer & Schulze, 2015; Hawa, Azhar, Top, & Zubaid, 2016; Mandal & Shankar Raman, 2016; Srinivas & Koh, 2016).

In major oil palm plantations and government‐controlled smallholdings, a stringent monoculture planting practice is implemented in order to maximize palm oil yield (Azhar, Saadun et al., 2015). For small‐scale farmers or smallholders, their farming system is not restricted to one type of commodity crop (Azhar et al., 2014), and polyculture is commonly practiced by small‐scale oil palm farmers in Southeast Asia. Little is known about the effect on biodiversity of polyculture in oil palm farming (Azhar et al., 2014). Smallholders usually grow different crop plants alongside oil palms for domestic consumption or sale. Crops such as banana, coconut, tapioca, and pineapple can be grown alongside oil palm trees (Azhar et al., 2014; Azhar, Puan et al., 2015). Such practice may increase habitat heterogeneity that is the key for maintaining biodiversity and also provide additional food sources in the case of fruit crops. The maintenance of faunal biodiversity in oil palm agriculture is mainly determined by multiple vegetation structure characteristics (Asmah et al., 2016; Azhar et al., 2014; Ghazali et al., 2016; Syafiq et al., 2016).

**Figure 1:**
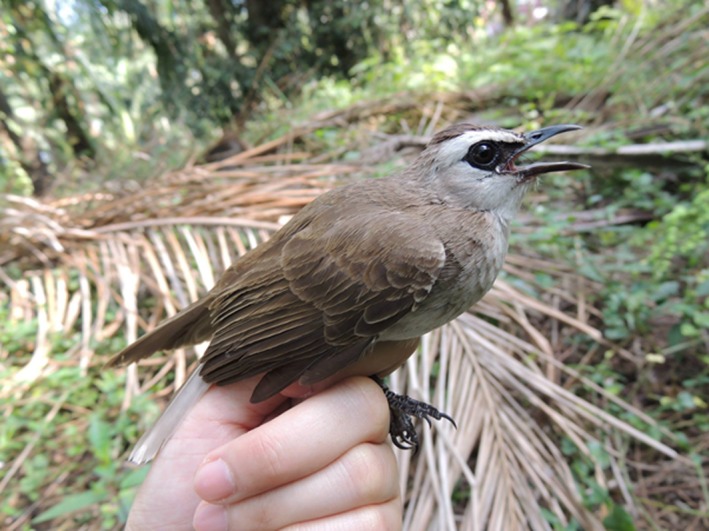
One of the common birds in oil palm production landscapes, the yellow‐vented bulbul (*Pycnonotus goiavier*)

The aim of this study is to shed new light on ways to enhance avian biodiversity in oil palm production landscapes (Figure 1). To date, the effort to transform conventional oil palm agriculture to a sustainable one is limited, and it has not determined what agricultural practices increase within‐plantation biodiversity. New research is required on developing practices that can improve biodiversity and compensate for the related loss of ecosystem functions (Dislich et al., 2016). This study addressed the following research questions: (1) Does bird species richness and abundance differ between polyculture and monoculture oil palm smallholdings? We predicted that polyculture plantations contain greater bird species richness and abundance than monoculture sites. (2) Within plantations, what are the most relevant in situ habitat quality characteristics that influence bird biodiversity? We predicted that bird biodiversity is influenced by key vegetation structure characteristics associated with oil palm agricultural practices such as understory vegetation cover and stand height.

## 2 MATERIALS AND METHODS

### 2.1 Study site

**Figure 2:**
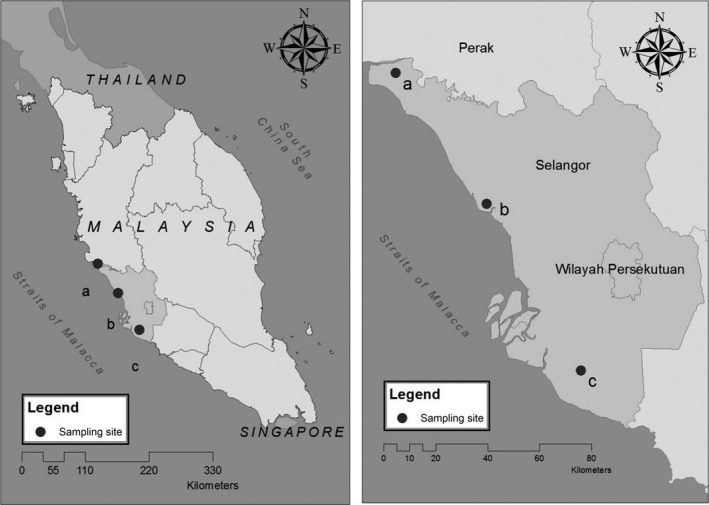
Map showing locations of the three study sites (a) Sabak Bernam, (b) Tanjung Karang, (c) Banting

We conducted this study at three areas within the state of Selangor, Peninsular Malaysia (Figure 2): Banting (2°46′42″N, 101°32′43″E), Tanjung Karang (3°22′34″N, 101°13′4″E), and Sabak Bernam (3°50′50″N, 100°52′0″E). These areas were predominantly oil palm plantations and managed by local farmers. Individual farmers usually owned plantations of less than 10 ha. The daily temperature ranges from 25°C to 31°C, and mean relative humidity is 65%–70%. Temperatures are highest from March to May, with the monsoon periods from October to December. Rainfall ranges from 60 to 340 mm per month over the last five years (Malaysian Meteorological Services Department 2014).

### 2.2 Sampling design

**Figure 3:**
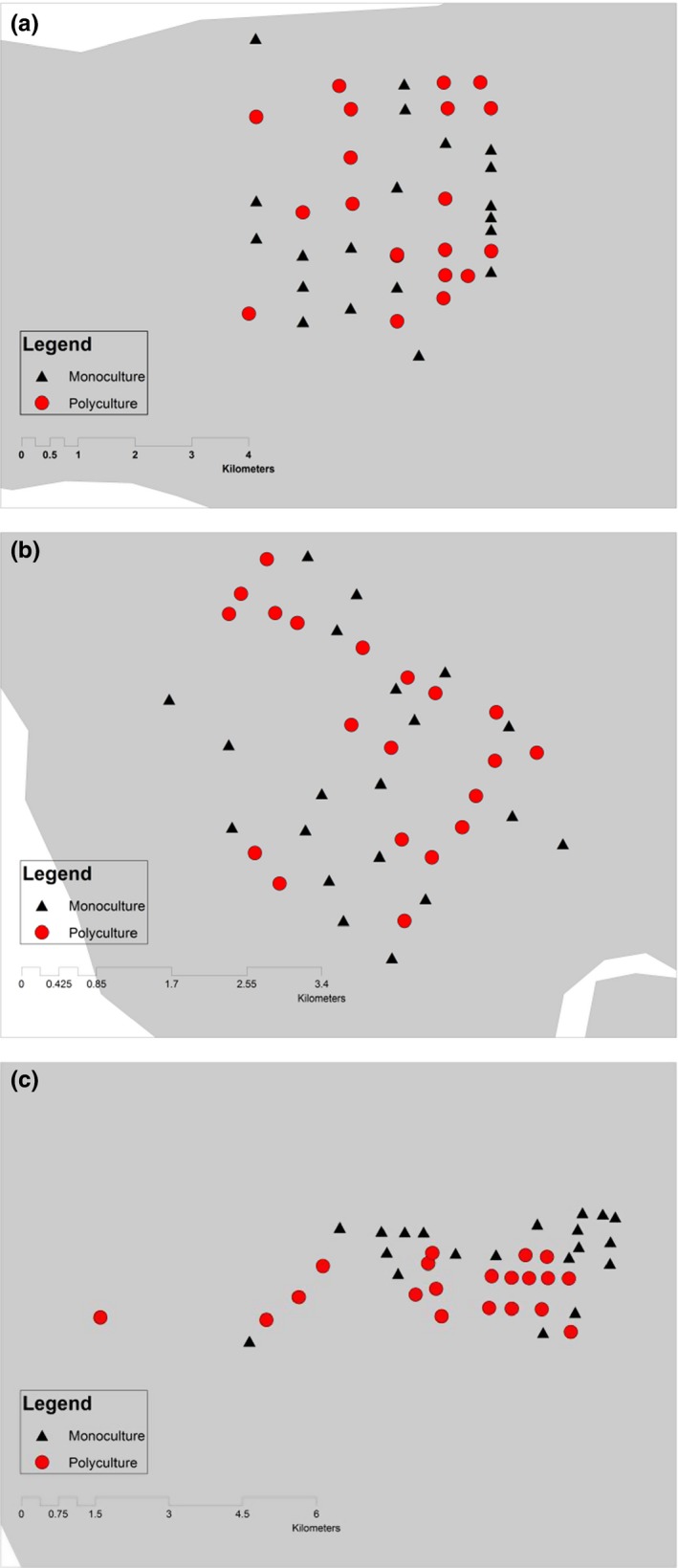
Map of study sites showing the 120 sampling points established within the oil palm smallholdings. (a) Sabak Bernam, (b) Tanjung Karang, (c) Banting

Our study area covered more than 18,000 ha of oil palm smallholdings (Figure 1). Each smallholding was less than 4 ha and owned by local villagers. The ages of oil palm stands at the sites varied from between 2 and 35 years. We grouped the points into two categories: (1) monoculture smallholdings planted only with oil palm (Figure 3), and (2) polyculture smallholdings where oil palm trees were planted with other crops, including banana trees and, less frequently, maize and tapioca (Figure 2).

We used a systematic sampling approach with random starting points (Morrison et al., 2008). We surveyed 120 sampling points, with 60 sites each in polyculture and monoculture smallholdings from February to April and from June to August in 2014. Sampling at each site was not repeated. We randomized visits to polyculture and monoculture smallholdings.

### 2.3 Bird sampling

We used mist nets (size: 9 m × 3 m) to sample birds. The mist nets were set up 1 m above the ground. At each site, two mist nets were concurrently set up 100 m apart for 96 mist net hr. The mist nets were checked every 20 min (according to the British Trust for Ornithology guidelines). After extraction and identification, birds were marked to allow for assessment of recaptures, birds were then released at the location where they were caught. Mist nets were open between 0,700 and 1,900 every day, and each mist net was in place for four days in total. Sites were at least 500 m apart to maintain sampling independence (Ralph, Sauer, & Droege, 1995).

### 2.4 Habitat quality measurement

We recorded the following vegetation characteristics within 100 m × 100 m plots situated on the harvesting path at each sampling point: (1) grass cover from four quadrats (1 m × 1 m); (2) nongrass cover (e.g., ferns and shrubs) from the quadrats; (3) height of grass cover from the quadrats; (4) height of nongrass cover from the quadrats; (5) epiphyte cover on oil palm trunks from a vertical plot of 0.5 m × 1 m; (6) canopy cover was determined using a GRS densitometer; (7) number of oil palm (>5 year old); (8) number of immature (<5 year old) oil palm; (9) number of dead oil palm (standing); (10) number of dead oil palm (fallen); (11) mean height of oil palm trees (measured using a digital laser rangefinder, Nikon Aculon); and (12) presence (polyculture) or absence (monoculture) of banana trees (Table 2).

### 2.5 Data analysis

To assess the overall sampling effort, we compared the observed bird species richness with the Chao 1 classic estimator for species richness in Estimates version 9.1 (Colwell, Mao, & Chang, 2004).

To evaluate for possible differences in sampling adequacy in the two structurally different habitats (i.e., polyculture and monoculture smallholdings), we produced expected species accumulation curves using the EstimateS package (Colwell, 2013). We performed two‐sample *t* tests to assess the difference between observed and estimated species richness.

We performed a one‐way analysis of variance (ANOVA) to compare species richness and abundance between the two different site categories (polyculture vs monoculture). The day of sampling (e.g., first visit and second visit) was used as a random factor in ANOVA. In addition, we contrasted habitat quality characteristics between polyculture and monoculture smallholdings by repeating the ANOVA procedures. Analyses of similarity (ANOSIM) were used to examine the differences in bird community structure between polyculture and monoculture sites in smallholdings. We ran permutation tests 999 times to obtain the inferential result.

To examine the relationships between cumulative species richness and habitat quality characteristics, generalized linear models (GLMs) were used. Poisson distribution and log‐link function were used in the modeling process. To select the final model, we used all possible methods (i.e., fitting of all possible regression models). We repeated the same procedures to analyze bird abundance. We conducted correlation tests to detect multicollinearity among predictor variables. Strongly correlated variables (|*r*| > .7) were dropped to avoid distortion in model estimation (Dormann et al., 2013). Only one variable from the correlated pair with lowest Wald statistic value was removed from the modeling process. Twelve predictor variables were tested in these analyses, from which two predictor variables were excluded because of multicolinearity. These include the number of mature oil palms (*r* = .753) in the species richness model and the number of mature oil palms (*r* = .725) and height of grass cover (*r* = −.7) in the abundance model. This analysis was performed in GenStat version 12.

BIOENV analysis was conducted to examine the relationship between bird assemblages and in situ habitat quality. We transformed (log base 10) and normalized environmental data. Bray–Curtis similarity and Euclidean distance similarity were used to create resemblance matrices for the abundance data and environmental data, respectively. We used Spearman rank correlation to identify the most important explanatory variables (Clarke & Gorley, 2006). We performed a permutation test 99 times to examine the relationship between species composition and a specific subset of explanatory variables. This analysis was performed in PRIMER version 6.

The spatial autocorrelation in residuals was examined by calculating Global Moran's Index in the ArcGIS^™^ version 10.1 (ESRI). We used the *p* value to reject or accept the null hypothesis, which states that the analyzed attribute is randomly distributed among the features in the study area (Mitchell, 2005). Inverse distance (nearby neighboring features have a larger influence on the computations for a target feature than features that are far away) was used to compute Global Moran's Index. We used Euclidean distance (the straight‐line distance between two points) as the distance method.

## 3 RESULTS

### 3.1 Species richness, abundance, and assemblages

A total of 865 birds from 42 bird species were captured during the study. The majority of bird species (31 species) were generally considered open country species rather than forest species (11 species). In terms of diet, the birds were mainly insectivores or carnivores (21 species), followed by granivores (seven species), omnivores (five species), nectarivores (five species), and frugivores (four species).

**Figure 4:**
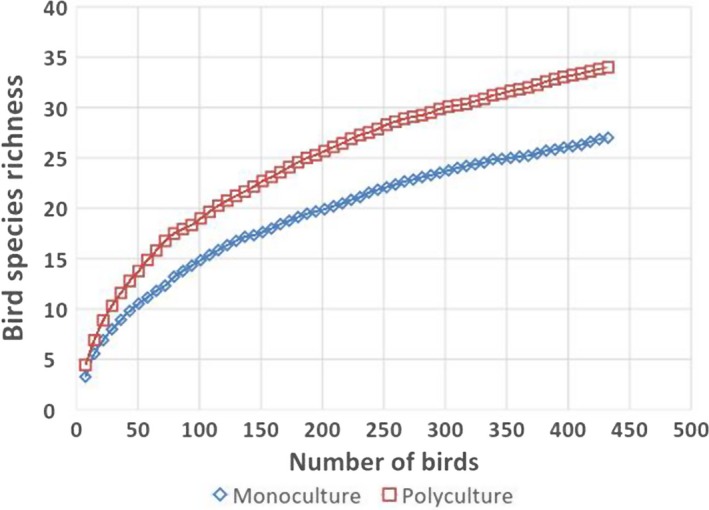
Species accumulation curves, with the *x*‐axis showing the number of individuals sampled. Bird species richness was higher in polyculture smallholdings than in monoculture smallholdings

Our mist netting effort yielded 52.8% of the true species richness. Our results revealed that there is a significant difference between observed and estimated species richness (*df *= 119, *t* = −127.99, *p* < .001). Similarly, observed species richness differed significantly from estimated species richness in both polyculture (*df* = 59, *t* = −29.60, *p* < .001) and monoculture (*df* = 59, *t* = −36.60, *p* < .001) sites. Based on species accumulation curve, bird species richness was greater in polyculture sites compared to monoculture sites at the same number of birds captured in oil palm smallholdings (Figure 4).

We found that the richness of birds was significantly greater (*F*_1,119_ = 5.88; *p *=* *.017) in polyculture sites ($\overset{¯}{x}$ = 3.78 species) than monoculture sites ($\overset{¯}{x}$ = 2.75 species). Variance ratio for random factors, that is different visits, was 4.40. Although bird abundance was higher in polyculture than monoculture, it was marginally nonsignificant (*F*_1,119_ = 3.43; *p *=* *.067) between polyculture sites ($\overset{¯}{x}$ = 6.76 birds) and monoculture sites ($\overset{¯}{x}$ = 4.93 birds). Variance ratio of the random factor was 13.68. With respect to habitat quality, only five of eleven characteristics were significantly different between polyculture and monoculture sites (Table 1).

**Table 1:** Comparison of 11 habitat quality characteristics between polyculture and monoculture sites in oil palm smallholdings

Habitat quality characteristic	Mean		*F*	*p*
	Polyculture	Monoculture		
Canopy cover (%)	58.2	73.7	9.60	.002
Epiphyte cover (%)	22.7	39.7	26.26	<.001
Grass cover (%)	30.4	31.2	0.03	.857
Nongrass cover (%)	31.3	26.7	1.52	.220
Height of grass (cm)	15.8	15.9	0.00	.969
Height of nongrass (cm)	23.6	22.6	0.17	.682
Height of oil palm stands (m)	6.68	9.07	25.63	<.001
Fallen dead oil palms	0.82	1.40	3.27	.073
Standing dead oil palms	0.400	0.633	1.48	.225
Number of immature oil palms	10.67	5.35	10.83	.001
Number of mature oil palms	13.13	20.68	27.46	<.001

Our results showed no significant difference in bird assemblages between polyculture sites and monoculture sites (Global *R* = 0.006; *p *=* *.22). Average similarity in assemblages among polyculture sites was 29.64% while among monoculture sites was 29.64%. Five species dominated more than 90% of the oil palm bird community at both polyculture and monoculture sites (Table 2).

**Table 2:** Bird species which contributed cumulatively > 80% of the bird community in polyculture and monoculture smallholdings

Smallholding type	Species	Guild	Average abundance per site	Contribution (%)	Cumulative (%)
Polyculture	Oriental magpie‐robin *Copsychus saularis*	Insectivore	1.02	32.08	32.08
	Yellow‐vented bulbul *Pycnonotus goiavier*	Frugivore	1.09	30.30	62.38
	Zebra dove *Geopelia striata*	Granivore	0.54	11.05	73.43
	White‐throated kingfisher *Halcyon smyrnensis*	Carnivore	0.60	10.27	83.70
	Common tailorbird *Orthotomus sutorius*	Insectivore	0.45	9.25	92.95
Monoculture	Oriental magpie‐robin *Copsychus saularis*	Insectivore	0.72	29.71	29.71
	Yellow‐vented bulbul *Pycnonotus goiavier*	Frugivore	0.82	27.29	57.00
	White‐throated kingfisher *Halcyon smyrnensis*	Carnivore	0.69	24.50	81.51
	Zebra dove *Geopelia striata*	Granivore	0.43	7.21	88.71
	Common tailorbird *Orthotomus sutorius*	Insectivore	0.43	7.21	88.71

### 3.2 Effects of in situ habitat quality on species richness, abundance, and assemblages

**Figure 5:**
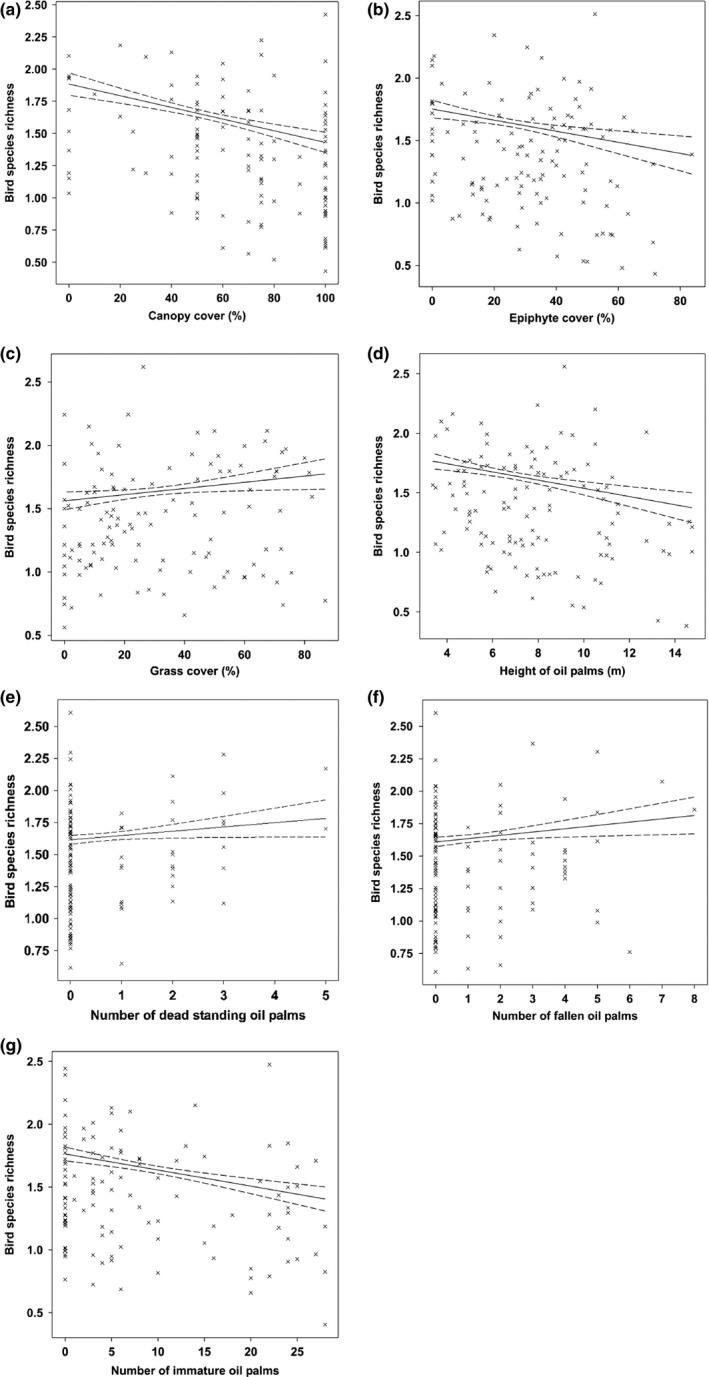
Scatterplots with 95% confidence intervals (dashed) on the regression (solid) line showing the relationships between the bird species richness and vegetation structure characteristics

Eight of the eleven explanatory variables explained 25.22% of the variation in bird species richness. Bird species richness increased with polyculture system, fallen dead oil palms, grass cover, and standing dead oil palms (Figure 5; Table 3). However, bird species richness decreased with canopy cover, number of immature oil palms, height of oil palm stands, and epiphyte cover (Figure 5; Table 3). We did not detect any significant effects from the height of grass, height of nongrass, and nongrass cover.

**Table 3:** Factors significantly influencing bird species richness in 120 sampling points located in oil palm smallholdings, modeled as a function of stand‐level attributes

Explanatory variables	Slope	*SE*	Wald statistic	*p*
Constant	2.2814	.0778	NA	NA
Canopy cover	−0.004536	.000787	33.22	<.001
Number of immature oil palms	−0.01280	.00250	26.18	<.001
Height of oil palm stands	−0.03443	.00802	18.44	<.001
Epiphyte cover	−0.00444	.00128	12.02	<.001
Polyculture system	0.1105	.0366	9.12	.003
Fallen dead oil palms	0.0255	.0103	6.14	.013
Grass cover	0.00244	.00105	5.39	.020
Standing dead oil palms	0.0334	.0165	4.08	.043

NA, not applicable; *SE*, standed error.

**Figure 6:**
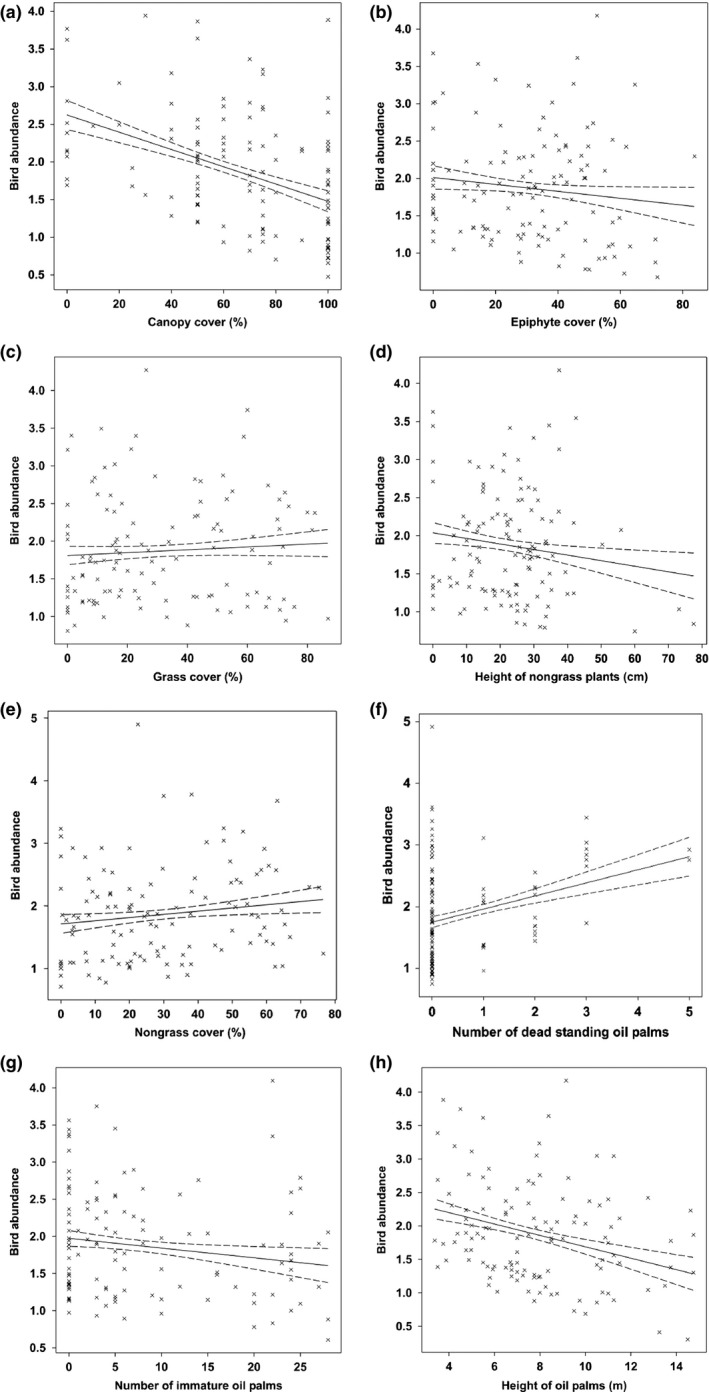
Scatterplots with 95% confidence intervals (dashed) on the regression (solid) line showing the relationships between the bird abundance and vegetation structure characteristics

Similar to bird species richness, eight of ten explanatory variables explained 29.36% of the variation in bird abundance. Bird abundance increased with standing dead oil palms, nongrass cover, and grass cover (Figure 6; Table 4). However, bird abundance decreased with canopy cover, height of oil palm stands, height of nongrass, epiphyte cover, and number of immature oil palms (Figure 6; Table 4). No significant effect from fallen dead oil palms and agricultural practice was detected.

**Table 4:** Factors significantly influencing bird abundance in 120 sampling points located in oil palm smallholdings, modeled as a function of stand‐level attributes

Explanatory variables	Slope	*SE*	Wald statistic	*p*
Constant	3.697	.202	NA	NA
Canopy cover	−0.01104	.00158	48.62	<.001
Standing dead oil palms	0.2112	.0366	33.33	<.001
Height of oil palm stands	−0.0991	.0174	32.40	<.001
Height of nongrass	−0.01304	.00350	13.92	<.001
Epiphyte cover	−0.00794	.00261	9.28	.002
Number of immature oil palms	−0.01430	.00545	6.89	.009
Nongrass cover	0.00511	.00216	5.60	.018
Grass cover	0.00356	.00166	4.60	.032

NA, not applicable; *SE*, standed error.

Bird assemblages were influenced by grass cover, height of nongrass vegetation, canopy cover, and number of banana plants. However, the permutation test showed that the inferential results were not significant (Rho = 0.114; *p *=* *.26).

We found that the spatial distribution of residuals was the result of random spatial process (monoculture system Moran's Index = −0.057786; *z‐*score = −0.790800; *p* = .429061; polyculture system Moran's Index = 0.019261; *z*‐score = 0.287112; *p* = .774026).

## 4 DISCUSSION

Our findings confirm that polyculture oil palm agriculture supports a greater bird species richness than monoculture oil palm. Large‐scale oil palm plantations or smallholdings that practice monoculture farming are characterized by homogenous vegetation and stand structure. In contrast, polyculture farming increases floristic diversity and stand structural complexity. As a result, it probably provides greater resources (e.g., food plants, prey availability, and shelter) for birds and other fauna than monoculture plantations (Jones & Sieving, 2006; Malézieux et al., 2009). In Sumatra, home gardens (which resemble the polyculture system (De Clerck & Negreros‐Castillo, 2000)) were found to be populated by omnivores and granivores, and frugivorous birds that were absent from monoculture oil palm (Prabowo et al., 2016). Intercropping oil palm with other edible plants or crops can also improve food security and cushion farmers from commodity price fluctuation (Koczberski & Curry, 2005; Koh, Levang, & Ghazoul, 2009). We suggest that polyculture farming is compatible with biodiversity and conservation strategies to make conventional oil palm agriculture more sustainable as well as benefiting smallholders financially. However, the polyculture smallholdings we sampled are limited to wider‐habitat bird species and will benefit a small number of specialized forest species. We suggest that additional benefits to avian biodiversity might be realized with the planting of native fruit trees that further increase stand structural heterogeneity and food resources. In addition, the opening of the canopy caused by the lower density oil palms may contribute to the increase in richness and abundance of birds in polyculture systems. The decrease in canopy cover can lead to a more substantial understory, which can provide favorable habitat for understory foraging or nesting birds (Azhar et al., 2011, 2013; Tejeda‐Cruz & Sutherland, 2004).

Within plantations, management is also important in maintaining biodiversity: Our results show that retaining dead oil palms in the plantation (both fallen and standing) increases bird species richness. It is likely that fallen, dead trees increase the abundance of decomposer insects such as saproxylic beetles, termites, and ants on the decaying oil palms, which provide food for birds (Seibold et al., 2015). Similarly in the forest environment, dead overstory trees can be important for forest biodiversity (Gibbons & Lindenmayer, 2002) and influence the rate of postdisturbance recovery processes (Franklin et al., 2000). Standing, dead trees can also be used by birds for nesting or as a base for aerial foraging (Guby et al., 1996), anecdotally; this behavior was witnessed by researchers during the sampling within plantations. The removal of these key structural attributes from the habitat may adversely influence species closely associated with them (Lindenmayer & Noss, 2006), thus substantially changing assemblages (Morissette, Cobb, Brigham, & James, 2002). We suggest that oil palm managers retain dead palm trees within the plantation to provide nesting and foraging habitat for birds, therefore increasing avian biodiversity. Retaining dead trees can also provide other benefits such as providing soil nutrients and carbon content when being decomposed for the plantation soil (Hughes, Kauffman, & Jaramillo, 1999; Montagnini & Nair, 2004). The downside of retaining dead palms is they can potentially harbor diseases such as white rot fungus, *Ganoderma boninense*. Control measures such as using antagonistic fungi, applying chemical treatments, and planting legume cover crops have been used to control *G. boninense* (Hushiarian, Yusof, & Dutse, 2013).

Polyculture practice similar to mixed‐species production stands can result in an increase in biodiversity (Felton, Lindbladh, Brunet, & Fritz, 2010). However, findings from a previous study in oil palm agricultural lands (Azhar et al., 2014) indicated that bird species richness was significantly greater in monoculture smallholdings than in polyculture smallholdings, but the opposite was true for bird abundance. The abundances of insectivorous and frugivorous birds were greater in polyculture smallholdings than in monoculture smallholdings (Azhar et al., 2014). The discrepancy in terms of results between the current study and (Azhar et al., 2014) is likely to be attributed to different sampling methods used in both studies. Point count method was used by (Azhar et al., 2014) which may record more bird species than those from mist netting.

Our results reveal that as oil palms get taller with age, bird species richness, and abundance decrease. The majority of previous bird studies in oil palm smallholdings did not account for oil palm height (Azhar et al., 2011, 2013, 2014) except one (Azhar, Puan et al., 2015), which did not find oil palm height to have a significant influence on bird species richness. A possible explanation for lower bird species richness and abundance with increasing cover and oil palm height is due to a sampling bias. The bias was apparently not seen in another study (Azhar et al., 2014), where a different sampling method (i.e., point transect) has led to different results. Our findings may be due to the sampling technique (i.e., mist nets) used in the current study. In areas with taller trees, birds visiting the oil palm canopy may be less likely to get caught in the mist nets and therefore not be sampled adequately. Bird species richness decreased with increasing canopy cover similar to the findings of (Azhar et al. 2011). Similarly, overall bird abundance decreased with increasing canopy cover; ground vegetation can be low in heavily shaded plantations, however, this is often caused by the heavy usage of herbicides. The lack of lower and middle story cover provides poorer conditions for foraging birds (McWethy, Hansen, & Verschuyl, 2010). Limited sunlight availability as the result of increasing canopy cover is often attributed to a decrease in understory species richness and abundance. Most bird, feeding guilds decrease in abundance with increasing canopy cover as (Azhar et al., 2013).

Bird species richness also decreased with the number of immature palms (less than five years). This may be because immature oil palms are less attractive to birds, perhaps because of limited resources (e.g., food and shelter), compared to more mature palms. For example, the trunks of young oil palms are less likely to support epiphytes that host arthropods (Fayle et al., 2010). In addition, mature oil palms occupy greater area than young palms and are able to provide more variable microhabitats for a wider range of species. Older oil palm stands also have more stable and cooler microclimate than young oil palm stands, which may be more favorable for birds (Luskin & Potts, 2011).

Our results suggest that the presence of some undergrowth vegetation features (i.e., grass cover) were significantly associated with bird richness and/or abundance. For instance, the presence of grass cover in oil palm smallholdings was found to positively and significantly influence both bird species richness and abundance. Similarly, the presence of nongrass cover was positively and significantly related to bird species richness. Our results are similar to another study conducted in an oil palm plantation in Guatemala, where removal of understory vegetation decreased bird richness (Nájera & Simonetti, 2010). The presence of understory in oil palm plantations therefore does appear to promote bird richness and abundance, perhaps by providing food resources and breeding sites for some species (Aratrakorn et al., 2006; Azhar et al., 2013). Management to maintain undergrowth, while still allowing easy access for harvesting, may therefore represent a key management practice to increase bird diversity as well as yielding other benefits, such as reducing soil erosion and improving soil invertebrate richness and abundance (Carron et al., 2015).

We found that bird species richness and abundance decreased slightly with epiphyte cover. This finding is contradictory to Prescott, Edwards, and Foster (2015) who found that removal of epiphytes did not affect the species richness and community composition of birds and ants in oil palm plantations. As epiphytes can determine the microclimatic conditions in their local areas and create a more stable temperature for insect communities (Turner & Foster,^2006^) that provide birds with animal protein. However, this may benefit a small number of gleaning insectivores that can seek insects on the epiphytes but not the whole insectivorous group.

## 5 CONCLUSIONS AND POLICY RECOMMENDATIONS

Tropical agricultural agrosystems such as polyculture smallholdings could be an important component of the conservation strategy in human‐modified landscapes as polyculture systems are able to support higher levels of biodiversity than monoculture plantations (Gardner et al., 2009; Perfecto & Vandermeer, 2008). In addition, oil palm agriculture is dominant in landscape matrices that enclose most protected forest reserves in Southeast Asia (Azhar, Saadun et al., 2015). With polyculture farming, oil palm smallholdings are likely to provide a higher quality habitat matrix that can permit the movement of forest organisms among patches of natural vegetation (Azhar et al., 2011; Azhar, Puan et al., 2015; Linkie et al., 2003). These agricultural practices may synergistically minimize adverse effects of simplified ecosystems like those found in oil palm production landscapes (Azhar et al., 2014; Ghazali et al., 2016; Syafiq et al., 2016). Both large protected areas (i.e., land sparing) and wildlife‐friendly agricultural matrices (i.e., land sharing) are needed to promote biodiversity conservation; they work synergistically and are not mutually exclusive (Kremen, 2015).

Existing monoculture oil palm landscapes are pervasive in producing countries such as Indonesia and Malaysia. Without changes in current farming practices, the ability of such production landscapes to sustain biodiversity will remain low, with only a few principal bird species supported in areas where large‐scale oil palm plantations persist. In addition, to retaining forest patches and riparian habitats beneficial for biodiversity (Gray et al., 2014; Lucey et al., 2014), stakeholders should be encouraged to practice polyculture farming as a means to improve biodiversity within plantations and to maintain important ecosystem functions such as biological pest control and pollination. The findings from this study can guide policy makers and certification bodies (e.g., Roundtable on Sustainable Palm Oil) to promote oil palm production landscapes that are managed more sustainably and improve local and regional biodiversity.

## CONFLICT OF INTEREST

None declared.
